# Between Resistance and Complicity, the Australian Healthcare Community and 30 Years of Immigration Detention

**DOI:** 10.1007/s10903-022-01389-7

**Published:** 2022-08-05

**Authors:** Ryan Essex, Erika Kalocsányiová

**Affiliations:** Institute for Lifecourse Development, The University of Greenwich, Old Royal Naval College, Park Row, Greenwich, SE10 9LS London, United Kingdom

**Keywords:** Refugee, Asylum seekers, Immigration detention, Health, Healthcare, Australia

## Abstract

This year marks 30 years since Australia introduced its policy of mandatory, indefinite immigration detention. We provide an overview of these policies with a focus on the involvement of healthcare workers, both within centres and externally, protesting these policies. We discuss several lessons that can be learnt from Australia?s approach, namely that traditional approaches to health and healthcare have done little to address the suffering of those who are detained. We call for the healthcare community to consider their role in activism and in calling for the abolition of detention. These lessons sadly have increasing global relevance with several countries now seeking to emulate Australia?s cruelty.

This year marks 30 years since immigration detention was introduced by the Australian government. Originally introduced as an “interim measure” to address the “pressing requirements of the current situation” [1], these policies have been maintained and expanded. Today, not only does the Australian government have substantial powers to detain anyone without a visa indefinitely, the main intent of these policies has turned to one of deterrence. That is, while the Australian government arguably uses detention for other ‘administrative’ purposes, these policies are mainly focused on deterring ‘unauthorised’ arrivals, and almost exclusively unauthorised arrivals by boat. This is best reflected in the policies of offshore immigration detention on Manus Island and Nauru, which were originally introduced in 2001–2008 and have been in place since 2013. Many who arrived in 2013 and who were sent offshore have been detained for over eight years. Similar stories can be found onshore, with some refugees detained for over a decade [2].

Over 30 years these policies have rarely left the headlines and have been an ever present issue in Australian politics. Here we find a number of constants; persistent reports of distress, violence and resistance have emerged from centres. Almost two decades ago the Australian Human Rights Commission (AHRC) shed light on the plight of detained children in the Last Resort report [3]. The People’s Inquiry into Immigration Detention [4] re-iterated a number of these issues and gave insight into what it was like to be detained in Baxter and Woomera Immigration Detention Centres; centres which have been long shuttered. In this report several health workers testified about the impact of the detention environment, which is perhaps best summed by a mental health professional who noted that “[y]ou couldn’t really design an environment more destructive to child development than immigration detention” (p. 49). The most recent AHRC report, the Forgotten Children makes for similar reading [5], this investigation found that immigration detention was having “profound negative impacts on the mental and emotional health of children” (p. 29). These reports have been bolstered by a growing body of evidence which has left no doubt about the devastating impact of these policies [6].

Beyond the suffering of children, Australian immigration detention has had a profound impact on all who have come into contact with these policies [7]. That is, these policies have resulted in the prolonged detention of individuals who are often traumatised and have already faced substantial adversity. Over the last three decades we have seen this manifest in a number of ways, in addition to remarkably high rates of psychological distress [8], despair and a range of other behaviours, such as self-harm. A recent study has put rates of self-harm and suicidal behaviour to be at least 216 times than that found in the Australian community [9]. In addition to this, offshore processing has raised several distinct issues related to healthcare. While the issue of transparency and accountability, along with the extent of healthcare services available have long been issues onshore, these concerns are amplified offshore with limited specialist services resulting in the need to transfer unwell patients to Australia, with at least one death attributed to a delayed transfer [10].

As alarming as the impact of detention has been the Australian government’s unwillingness to entertain alternatives to this approach, despite these harms being long and well known [2]. In doing this, the Australian government has been belligerent, either ignoring or dismissing the above evidence, or even attacking those who have advocated for a shift in policy.

The Australian healthcare community has been closely involved with immigration detention since it was first introduced, providing services within centres. The position of the healthcare community could be seen as one that exists between complicity and resistance, raising a number of intractable ethical issues. On the one hand, healthcare provided within detention is largely futile, failing to buffer against the harms of detention. In this respect healthcare workers have been central to the system’s function; the system could not exist how it has without healthcare being provided within detention centres [11]. Several former clinicians have spoke of their time working in detention. Dr Nick Martin, a GP reflected on his despair in trying to change the system while on Nauru, writing, “I felt a hollow desperation. I was stuck, and needed to change the script. How to change things? How to get people to do something?” [12]. This position has not gone unrecognised by others, with detainees speaking out about the compromised and almost futile nature of healthcare within detention [13].

This however is not the end of the story. Outside of centres healthcare workers and their professional bodies have called for change [14], they have led research that has detailed the impact of detention and formed the basis for greater advocacy, some of which we have outlined above. Australian immigration detention has also led to other forms of action that were, when they came to healthcare workers in Australia, unprecedented. Clinicians have marched, whistleblown and even engaged in civil disobedience in opposition to immigration detention, these acts have been largely supported by professional healthcare bodies [2]. Many of these acts have also had substantial impact. The Broder Force Act, legislation which criminalised disclosures about working within immigration detention centres was quietly repealed after multiple healthcare workers either broke or threatened to break this law, persistent protest from the broader healthcare community, and a looming High Court challenge [15]. Despite years of defiance and attacks from the government, children were released from onshore immigration detention in 2015 (this is of course a generous assessment, given children still remain in detention, but in smaller numbers) [16]. This would not have occurred without the AHRC Forgotten Children report and persistent efforts from clinicians, highlighting the harms of detention and advocating for the release of children. More recently, the ‘Medevac’ legislation would not have been passed if not for persistent agitation from the healthcare community. While short lived, the brief period while this legislation was in effect resulted in 192 transfers to Australia for medical treatment [17], more recently and again after persistent pressure, the majority of those transferred to Australia under this legislation were released to the community [18].

Australia has arguably led the world in cruelty when it comes to immigration detention, there are however several lessons that can be taken away when it comes to health and healthcare. Today these lessons sadly have particular relevance for the international community, with several countries already processing asylum seekers offshore [19] or seeking to emulate Australia’s approach [20]. First, evidence and appeals to humanity mean little when there are greater political motives at play. That is, the harms of detention have been well established and the multiple reasons given for the necessity of these policies have long been debunked. This however has done little, with Australia’s approach only becoming increasingly punitive over the last three decades. The implication of this is that we need to look to strategies that impose costs on the Australian government and others who benefit from these policies. From the small number of victories above, strategies and actions that have sought to make these policies unsustainable (politically, socially, economically) have been the most effective. Second, detention has a devastating impact on health and this cannot be addressed while people remain detained. In many ways, it makes little sense to speak about the provision of healthcare within detention. The aim for those concerned about these policies and the health of those detained should be abolition, or at the very least, substantive reform. Third, and following these points, resistance has been an effective means to pressure the Australian government and in some cases, has led to small positive changes. While the tactics of social movements are hotly debated and while there is no formula we can turn to that guarantees success, in the case of Australian immigration detention we can see how health and healthcare have been utilised as central reasons to reform these policies, we can see how the healthcare community have leveraged their expertise and built alliances with others, such as lawyers and journalists, we can learn from episodes where opportunities have been exploited (see Medevac above), we can also see the impact of disruptive actions in demanding change. The point is, while more research on the harms of detention is needed, there is just as pressing a need to explore how substantive political change could be achieved in this space. Finally, there is also a need to look toward longer change, while far fewer people are detained in both onshore and offshore than a decade ago, the Australian government holds substantial power to detain anyone who travels to Australia without a valid visa. A plan for proactive, longer term change is needed to counter dominant narratives about refugees and asylum seekers and educate the Australian public about the impact of these policies. Looking ahead, we face a multitude of challenges such as climate change and growing inequality. We should heed the lessons that we can take from Australian immigration detention, in how we support he most vulnerable in the face of oppression and deliberate harm. We should continue to question how we may be complicit in such systems and importantly, how they can be resisted.

Note: Ryan Essex worked in Australian immigration detention centres for four years as a Counsellor and has spent the last decade researching their impact. Erika Kalocsányiová has researched issues impacting refugees’ integration processes and outcomes, among them the health and mental health impacts of immigration detention.
